# Group B *streptococcus *serotype prevalence in reproductive-age women at a tertiary care military medical center relative to global serotype distribution

**DOI:** 10.1186/1471-2334-10-336

**Published:** 2010-11-24

**Authors:** Danielle L Ippolito, Wesley A James, Deborah Tinnemore, Raywin R Huang, Mary J Dehart, Julie Williams, Mark A Wingerd, Samandra T Demons

**Affiliations:** 1 Department of Clinical Investigation, Madigan Healthcare System, 9040 Reid St., Tacoma, WA 98431 USA; 2 Department of Pathology, Madigan Healthcare System, 9040 Reid St., Tacoma, WA 98431 USA

## Abstract

**Background:**

Group B *Streptococcus *(GBS) serotype (Ia, Ib, II-IX) correlates with pathogen virulence and clinical prognosis. Epidemiological studies of seroprevalence are an important metric for determining the proportion of serotypes in a given population. The purpose of this study was to evaluate the prevalence of individual GBS serotypes at Madigan Healthcare System (Madigan), the largest military tertiary healthcare facility in the Pacific Northwestern United States, and to compare seroprevalences with international locations.

**Methods:**

To determine serotype distribution at Madigan, we obtained GBS isolates from standard-of-care anogenital swabs from 207 women of indeterminate gravidity between ages 18-40 during a five month interval. Serotype was determined using a recently described molecular method of polymerase chain reaction by capsular polysaccharide synthesis (cps) genes associated with pathogen virulence.

**Results:**

Serotypes Ia, III, and V were the most prevalent (28%, 27%, and 17%, respectively). A systematic review of global GBS seroprevalence, meta-analysis, and statistical comparison revealed strikingly similar serodistibution at Madigan relative to civilian-sector populations in Canada and the United States. Serotype Ia was the only serotype consistently higher in North American populations relative to other geographic regions (p < 0.005). The number of non-typeable isolates was significantly lower in the study (p < 0.005).

**Conclusion:**

This study establishes PCR-based serotyping as a viable strategy for GBS epidemiological surveillance. Our results suggest that GBS seroprevalence remains stable in North America over the past two decades.

## Background

*Streptococcus agalactiae *(Group B *Streptococcus *[GBS]) was first identified as a significant public health concern in maternal fetal medicine in the 1970s. Since this time, more than 7500 cases of GBS-associated neonatal sepsis and meningitis have been reported annually, with a financial burden of more than $350 million per year in neonatal costs [1]. Population-specific surveillance studies provide an important vehicle for evaluating the public health risks posed by changes in distribution of GBS serotypes [2].

Anogenital colonization with GBS is usually asymptomatic in immunocompetent adults [3]. Perinatal transmission of GBS from infected mothers during delivery can cause potentially fatal sepsis and meningitis [4]. Classification of GBS serotype is based on 10 immunologically unique capsular polysaccharides associated with pathogen virulence and encoded in the capsular gene cluster (*cps*) (Ia, Ib, II-IX) [5]. In the United States civilian population the predominant GBS serotypes are Ia, III, Ib, and V. Current multivalent vaccine development depends on accurate population data of serotype distribution [6]. Thus, epidemiological studies of seroprevalence are important in assessing changes in GBS distribution.

Accurate epidemiological characterization of GBS isolates is dependent on unequivocal classification of serotypes. Molecular methods have improved the accuracy and feasibility of serotyping, but these methods have only recently been applied to large cohorts [7].

Global serotyping distribution studies have documented that the prevalence of a given GBS serotype varies according to geographical location and time of study conductance [2]. Surveillance data in the United States come largely from civilian hospitals, with the Active Bacterial Core surveillance system within the Emerging Infections Program Network providing a mechanism for routine population-based surveillance [8]. Military communities are unique in their demographically diverse population including transients from military bases across the United States and around the world. It is unknown how the geographical mobility of the military might affect GBS seroprevalences in military cohorts.

Madigan Healthcare System (Madigan) is a tertiary care military medical center providing obstetric care to military families, with more than 2100 deliveries reported in 2009. Women between the ages of 18-40 are screened for GBS by urine cultures and vaginal/rectal swabs as standard of care. The objective of this study was to apply a two-tiered polymerase chain reaction (PCR) approach to determine the prevalence and distribution of the GBS serotypes in the Madigan population during the period covering July - December 2009 and to compare Madigan seroprevalences with global distributions.

## Methods

### Study population

This study was performed with the approval of the Madigan Healthcare System Institutional Review Board. Of the 1129 patients screened during the study period (July 2009 - December 2009), 207 were scored as GBS positive (18.3%). Clinical GBS isolates from these patients were obtained from vaginal/rectal swabs routinely conducted on women of indeterminate gravidity between ages 18-40. GBS isolates were disassociated from any personal health information and assigned a unique study number correlating with the date of specimen collection.

### Bacterial culture and banking

GBS isolates were cultured from vaginal/rectal swabs. GBS positive diagnoses were confirmed by conventional morphologic and phenotypic methods. Isolated GBS colonies were obtained taking vaginal/rectal swabs and placing them directly into 5 ml of Lim enrichment broth (Fisher Scientific, Pittsburgh, PA) and grown overnight at 35°C in an 8% CO_2 _incubator. One microliter loop of culture was quadrant streaked on blood neomycin plates (Fisher Scientific, Pittsburgh, PA) in order to obtain isolated colonies. Single colonies were then inoculated in 5 ml of LB broth and grown overnight under the same conditions stated above. Cultures (700 μl) were placed in 1.5 ml cryovial tubes with 300 μl of 50% glycerol to make glycerol stocks. Samples were then flash frozen with liquid nitrogen and stored at -80°C. Glycerol stocks were streaked overnight onto blood neomycin plates and colonies with a clear zone of hemolysis were selected for PCR amplification.

### Serotyping of GBS Isolates

Classification of GBS samples was done by PCR-based restriction fragment length polymorphism (RFLP) capsular typing to distinguish among nine of the ten known GBS serotypes (Ia, Ib, II-IX). We used PCR primers and conditions published by Manning *et al *(cpsG-F97, cpsL-R200, cpsR8-F40) [9]. These primers were used to amplify DNA fragments using Pure Taq Ready-to-Go PCR Beads (GE Healthcare, Pittsburgh, PA) followed by digestion with DdeI (New England Biolabs, Ipswich, MA) to generate restriction fragments. Digested amplicons were separated on 1.8% agarose gels and analyzed using a Typhoon 9410 variable mode imager (GE Healthcare, Piscataway, NJ). Serotype classification was determined by comparison of the molecular weight banding pattern of the resulting restriction fragment length polymorphisms (RFLPs) with published DNA fragment sizes of the GBS *cps *gene cluster [9].

### Serotyping by multiplex polymerase chain reaction

GBS-specific primers (DLTS) were used to identify those isolates that were nontypeable (NT) by RFLP analysis of the *cps *gene cluster. Multiplex PCR confirmed serotype classification and serotyped GBS isolates that yielded ambiguous results by the RFLP analysis of the *cps *gene cluster. Multiplex PCR was performed on single colonies using Pure Taq PCR Ready-to-Go PCR beads using the cps primers and PCR conditions published by Poyart *et al *[10]. Isolates classified as NT by both methods were serotyped using the primers and methods detailed in Imperi *et al *[7].

### Statistical analysis, systematic review, and multiple comparisons analysis

An *a priori *power analysis indicated that a minimum of 400 patients should be enrolled in the study, assuming a 20% rate of colonization with 5% error to determine prevalence with a precision (α) of 5% [11]. Based on this estimation, a total of 1129 patients were screened, of which 207 (18.3%) were determined to be GBS positive. To compare the Madigan cohort to the global serotype distributions, a systematic review was conducted of 426 primary literature references evaluating serotype distribution in discrete geographical regions. These studies were identified in a Pub Med keyword search for references reporting population serotyping data for GBS (through February 2010). Studies were excluded if GBS was obtained from normally sterile sites (i.e., blood, synovial fluid, etc.). Studies reporting serodistribution in neonates were likewise excluded. Of the papers surveying predominantly colonized women, we excluded studies predating the emergence of serotype V in the general population. Where raw numbers were available, we confirmed the investigator-reported serotype percentages by our own independent calculations. In two instances, the frequencies we calculated did not match the percentages reported in the article due to obvious typographical errors. We relied solely on data in article abstracts where the full text articles were not written in the English language or if the full text article could not be procured. Meta-data were compiled by calculating proportions of serotype distribution among all GBS-positive diagnoses segregated by geographical region (Table 1). Studies included in the global statistical analysis are presented in systematic review in Table 2 (United States and Canada) [12-26] and Table 3 (global data) [11,27-64]. The proportional z-test was used to determine significance between serotype rates of the Madigan cohort and other regions. The statistical significance level was stringently set by the Bonferroni correction method to be p < 0.005.

**Table 1:** Multiple comparisons analysis comparing the Madigan cohort with global GBS serotype distributions.

		Serotype Distribution (%)										
		
Region	Patients	Ia	Ib	II	III	IV	V	VI	VII	VIII	NT	
Madigan	207	59	25	25	56	4	35	2	0	0	1	**#patients**
		**28.50**	**12.08**	**12.08**	**27.05**	**1.93**	**16.91**	**0.97**	**0.00**	**0.00**	**0.48**	**%**

Canada	353	77	35	40	66	5	78			1	49	**#patients**
		**21.81**	**9.92**	**11.33**	**18.70**	**1.42**	**22.10**	**0.00**	**0.00**	**0.28**	**13.88**	**%**
		0.104	0.512	0.906	0.027	0.918	0.171				0.001	**p-value**

US	7609	2039	618	826	1887	73	1139	26	3	17	869	**#patients**
		**26.80**	**8.12**	**10.86**	**24.80**	**0.96**	**14.97**	**0.34**	**0.04**	**0.22**	**11.42**	**%**
		0.642	0.055	0.658	0.511	0.299	0.501	0.364			0.001	**p-value**

Europe	2662	485	329	384	747	98	396	16	16	15	131	**#patients**
		**18.22**	**12.36**	**14.43**	**28.06**	**3.68**	**14.88**	**0.60**	**0.60**	**0.56**	**4.92**	**%**
		0.001	0.93	0.407	0.817	0.265	0.492	0.847			0.006	**p-value**

ME, EEu	566	78	24	76	82	17	79	10	7	0	41	**#patients**
		**13.78**	**4.24**	**13.43**	**14.49**	**3.00**	**13.96**	**1.77**	**1.24**	**0.00**	**7.24**	**%**
		0.001	0.001	0.709	0.001	0.575	0.363	0.64			0.001	**p-value**

Asia	1521	171	214	132	446	25	266	81	22	48	85	**#patients**
		**11.24**	**14.07**	**8.68**	**29.32**	**1.64**	**17.49**	**5.33**	**1.45**	**3.16**	**5.59**	**%**
		0.001	0.502	0.142	0.553	0.987	0.913	0.01			0.003	**p-value**

Africa	260	37	12	22	89	10	82	0	0	0	7	**#patients**
		**14.23**	**4.62**	**8.46**	**34.23**	**3.85**	**31.54**	**0.00**	**0.00**	**0.00**	**2.69**	**%**
		0.001	0.005	0.256	0.118	0.349	0.001				0.142	**p-value**

S. Am	113	23	12	28	13	2	6				10	**#patients**
		**20.35**	**10.62**	**24.78**	**11.50**	**1.77**	**5.31**	**0.00**	**0.00**	**0.00**	**8.85**	**%**
		0.144	0.836	0.006	0.002	0.749	0.005				0.001	**p-value**

Mexico	492	277		112	88						16	**#patients**
		**56.30**	**0.00**	**22.76**	**17.89**	**0.00**	**0.00**	**0.00**	**0.00**	**0.00**	**3.25**	**%**
		0.001		0.002	0.009						0.057	**p-value**

Aus, NZ	125	25	24	5	26	4	37	0		0	2	**#patients**
		**20.00**	**19.20**	**4.00**	**20.80**	**3.20**	**29.60**	**0.00**	**0.00**	**0.00**	**1.60**	**%**
		0.111	0.107	0.022	0.251	0.728	0.01				0.655	**p-value**

NOTE. Bonferroni correction alpha = 0.05/p = 0.005 (two-sided); statistical significance < 0.005 relative to Madigan cohort

Madigan, Madigan Healthcare System; ME, Middle East; EEu, Eastern Europe; S.Am, South America; Aus, Australia; NZ,New Zealand

**Table 2:** United States and Canada GBS Seroprevalences Relative to Madigan Distribution

							Prevalence by Serotype (%)										
								
Location	Study Interval	Patient Population	Typing method	total # patients	% GBS+	# Total Iso-lates	**Ia**^**a**^	Ib	II	III	IV	V	VI	VII	VIII	NT	Ref
**WA (Madigan)**	**2010**	**Women**	**PCR**	**1129**	**18.3**	**207**	**29**	**12**	**12**	**27**	**2**	**17**	**1**	**0**	**0**	**1**	**this study**
Canada Alberta	1998-2000	Gravidas	OID	NA	NA	118	20	9	9	15	0	28			0	20	[15]
Canada Calgary	1998-2000	Gravidas	OID	1207	19.5	235	23	11	13	21	2	19			0.4	11	[15]
MA Boston	1999	>94% Women	ELISA	NA	NA	114	25	5	10	24	0	32	0		0.9	4	[23]
MA Boston	1962-1963	>90% Women	ELISA	NA	NA	149	46	14	11	22		0			0	7	[23]
MI	1997-2000	Adults	DB	NA	NA	338	21	12	13	13	1	22	2	0	0.3	12	[20]
MI	1999-2000	Gravidas	LCP	NA	NA	117	18	11	11	19	1	20	2			17	[19]
MI^c^	1999-2000	92% Adults	DB	NA	NA	306	23	12	14	11	3	30	3	0.7	2	1	[12]
MN	1974- 1975	3rd Trimester Gravidas	CP	802	5.6	45	26	13	24	24						13	[17]
MN	1993-1999	Adults	NA	NA	NA	138	21	14	16	5	0.7	35				9	[24]
OH	2001-2002	Gravidas, urine cultures	LCP	65	2.8	2318	23	4	4	32	2	6				30	[21]
OH	2003			NA	NA	349	18	9	11	17		27					[14]
PA Pittsburgh	1993-2002	non pregnant women	IP	NA	NA	2660	34	11	13	25	0.4	15	0.3	0	0.3	2	[16]
PA Pittsburgh	1998-2000	non pregnant women	DB	NA	NA	177^b^	31	9	15	28	0.6	14				3	[22,26]
TX	1994-1995	Gravidas	AS	546	28	153	24	9	26	24	0	12				4	[18]
WA, TX Seattle; Houston	1992-1995	Gravidas	ID	3307	26	856	26	8	18	21	0	21	0.2			1	[13]
WV	1999	Females	LA	NA	NA	84	19	13	9	15	4	15				9	[25]

a--Ic from earlier studies is grouped with Ia

b--of 270 GBS+ patients, 177 were serotyped

c--mixed population: 267 adults - Michigan, 24 neonates - Houston, TX

Madigan-Madigan Healthcare System, Tacoma, WA

LCP-Lancefield capillary precipitin

OID-Ouchterlony immunodiffusion

DBC-dotblot capsular typing

ID-immunodiffusion with rabbit antisera

IP-immunoprecipitation in agarose

LA-latex agglutination with serotype specific sera

NA-not available

AS-antisera

**Table 3:** Global GBS Serotype Distribution in Reproductive-Age Adults Relative to Madigan Healthcare System.

							Prevalence by Serotype (%)										
								
Location	Study Interval	Patients	Typing method	# Total Patients	% GBS+	# GBS+ Isolates	**Ia**^**a**^	Ib	II	III	IV	V	VI	VII	VIII	NT	Ref
**Madigan, USA**	**2010**	**Women**	**PCR**	**1129**	**18.3**	**207**	**29**	**12**	**12**	**27**	**2**	**17**	**1**	**0**	**0**	**0.5**	**this study**

**Europe**																	

Czech R	2001-2002	Gravidas	IP	586	29.4	172	22	~10	~13	33	0	14	~3			1.8	[46]
France^T^	2001	Gravidas	PCR	39	8	500	26	8	8	41		18					[60]
*Germany*	1997-1999	Gravidas	EA	NA	NA	146	18	8	15	29	3	13				14	[63]
*Germany*	2001-2003	Gravidas	OID	460	23	104(75)	17	15	21	28	3	16	0	0	0	0	[30]
*Greece*	2000-2001	Gravidas	LA	1014	6.6	67	19	12	27	22	3	9	3	3	2		[58]
Ireland	1998 pub	Women	NA	504	25.6	129	30		17	30	1	9					[40]
Ireland	1999-2001	Gravidas	IP	NA	NA	20	35	5	0	30	0	20	0	0	0	5	[11]
Ireland^D^	2003 pub	Gravida^c^	NA	NA	NA	159	20	19	11	30	2	15				4	[32]
*Italy*^*P*^	1993-1995	Gravidas	OID	2300	11.3	260	16	27	22	22	3	5	0	0	5		[52]
*Italy*^*T*^	2005-2006	Gravidas	LA	400	18	73	21	7	6	33	8	26					[51]
*Netherl*	1999-2004	Gravidas	LA	NA	NA	92	26	7	13	22	8	15	8				[61]
*Portugal*^*L*^	2002-2004	Gravidas	LA	NA	NA	269	16	5	17	22	2	22	0	2	0	14	[44]
Sweden	2005	Women	LA,PCR	1579	25.4	356	11	13	16	24	15	19	0.5	1	0	1	[38]
Sweden	1995-1996	Gravidas	LA, IP	NA	NA	114	13	13	11	32	3	22				6	[29]
*U.K.*	2001-2003	Gravidas	LA	748	21.3	159	26	16	9	26		19				2	[39]

**Eastern Europe/Middle East**																	

*Kuwait*	2004 pub	Gravidas	AS	847	14.6	124	13	2	8	24	1	22	8	6	0	17	[27]
Iran	2003	Gravidas	NA	110	9.1	1197											[49]
*Israel*	2000	Gravidas	AS	681	12.3	84	18	11	27	20		14					[43]
Lebanon	2006	Gravidas		775	17.7	137	15	7	11	16	1	23				29	[53]
*Turkey*	2000 2001	Gravidas	LA	500	9.2	54	26	4	29	19	2	0				20	[34]
UAE	1998-1999	Gravidas	ID	563	10.1	57	21		4	18	26	12				16	[28]

**Asia**																	

India^D^	1980s	Women	NA	NA	NA	110		20	40	30						10	[64]
India^V^	1980s	Women	NA	NA	NA	79	20	41	53	6							[64]
*Japan*	1999-2000	Gravidas	ELISA	48	8.2	583	8	13	8	10	0	6	19	0	27	8	[45]
*Japan*	1992-2001	Gravidas	IS				9	6	2	10		9	27		32	6	[56]
*Japan*	1992-1994	Gravidas	E, OID	441	16	71	7	8	0	11	0	4	25	0	36	10	[41]
*Korea*	2006-2008	Gravidas	LA	2624	8	352	12	10		44		20				2	[42]
*Korea*	1990-2000	Gravidas	LA	NA	NA	446	10	22	2	37	1	21	4	1	1	1	[59]
Malaysia	2008	Gravidas	LA	NA	NA	200	12	2	6	12	10	19	17	5	2	17	[31]
Myanmar^Y^	1999-2001	Gravidas	CP	226	7.1	14	14	0	36	0	0	36	0	0	0	14	[11]
Taiwan	2000-2005	Gravidas	LA	NA	NA	58	22	9	3	33	0	26	3				[61]
Phillip^M^	1999-2001	Gravidas	CP	200	7.5	15	7	7	27	33	0	13	0	0	7	7	[11]
Thailand^BK^	1999-2001	Gravidas	CP	400		52	17	2	13	17	0	27	0	15	0	8	[11]

**Africa**																	

Gambia	1998	Gravidas	AbTyp	136	19.9	27	19	4	26	7	4	37				4	[55]
Nigeria	Early 1980s	Women	NA	NA	NA	89		12	6	62						20	[64]
Zimbabwe^H^	1999-2001	Gravidas	CP	210	11.9	21	10	14	5	24	0	43	0	0	0	5	[11]
Zimbabwe	2000 end	Gravidas	AbTyp	206	31.6	65	11	3	1	42	3	37				2	[48]
Zimbabwe	2002 end	Gravidas	AbTyp			117	15	5	4	45	5	24				2	[47]

**South/Central America and Mexico**																	

Argentina	NA	Gravidas	LA	531	3.2	17											[57]
Brazil	2003-2004	Gravidas	AS	316	14.6	46	17	24	20	4	7	9				17	[54]
Mexico	2000	Gravidas	LA	946	13	123	59		30	6						6	[62]
Mexico	2000-2001	Women	LA			25	68		4	28							[62]
Mexico	1999-2001	Gravidas	LA	691	14	97	62		26	13							[36]
Mexico	1988-1998	Gravidas	LA			169	44		20	30						5	[50]
Mexico		Gravidas				78	68		19	13							[36]

**Australia/New Zealand**																	

N. Zea	1998-1999	Gravidas	ID; PCR	240	52	22	21	20	6	29		20	2		2		[37]
*Australia*	1991-1992	Adults^b^	LA			103	19	19	4	19	4	32				1.9	[33]
***Italicized countries host US military bases (source: Military.com at ***http://www.military.com/ )																	

a--Ic from earlier studies is grouped with Ia

b--genital/urine data only are compiled

c--70% Gravidas

Madigan--Madigan Healthcare System, Tacoma, WA

pub--study publication date; interval not given

LCP-Lancefield capillary precipitin

AbTyp--Antibody typing

OID-Ouchterlony immunodiffusion

DBC-dotblot capsular typing

ID-immunodiffusion with rabbit antisera

IP-immunoprecipitation in agarose

LA-latex agglutination with serotype specific sera

E--ELISA

NA-not available

EA-enzyme extraction

CP-capillary precipitation

Czech R--Czech Republic

France^T^--France (Tours)

Ireland^D^--Ireland (Dublin) Italy^P^--Italy (Perugia)

Italy^T^--Italy (Turin)

Netherl--Netherlands

Portugal^L^--Portugal (Lisbon)

UAE--United Arab Emirates

India^D^--India (Delhi)

India^V^--India (Vellore)

Myanmar^Y^--Myanmar (Yangon)

Philippines^M^--Philippines (Manila)

Thailand^BK^-- (Bankok, Kohn Kaen)

Zimbabwe^H^--Zimbabwe (Harare)

NZ--New Zealand

## Results

**Figure 1:**
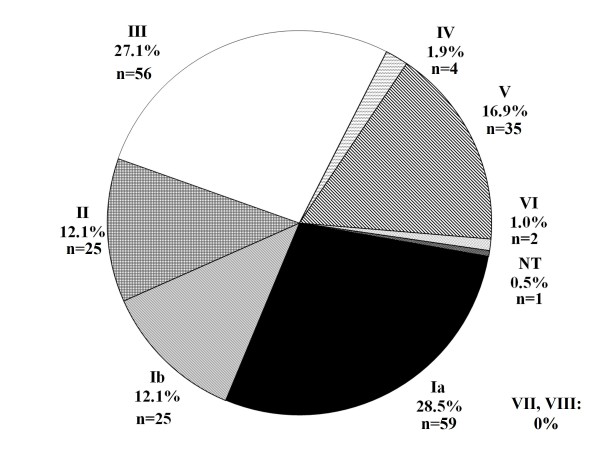
**Prevalence of GBS serotypes at Madigan Healthcare System July 2009 - December 2009**. Serotype was determined for GBS isolates collected from vaginal/rectal swabs from women of indeterminate gravidity at Madigan Healthcare System (Joint Base Lewis-McChord, Washington, USA). Percentages refer to serotype frequency in 207 total isolates screened. Total numbers of patients testing positive for each isolate are listed in parentheses. NT, non-typeable.

Four isolates were classified as NT by PCR amplification and DdeI restriction endonuclease digest alone. These isolates were confirmed to be GBS antigen B by GBS-specific primers dlts-F and dlts-R. Isolates were further screened by a multiplex approach to verify CPS serotype [10]. The single isolate not typeable by either method was further screened by GBS IX-specific primers [7], but results were negative. The serotype distribution results of the multi-tiered typing strategies are presented in Figure 1. Our results show that serotypes Ia, III, and V were the most abundant of the screened GBS isolates (28.5%, 27.1%, and 16.9%, respectively), followed by the less prevalent serotypes Ib, II, IV, and VI (12.1%, 12.1%, 1.9%, and 1.0%, respectively). GBS serotypes VII, VIII, and IX were not detected in our population group and one isolate was NT.

A systematic review of the literature compared Madigan seroprevalences with those reported within the rest of the United States (Table 2) and internationally (Table 3). Global proportions were combined by geographical region into a composite multiple comparisons analysis to compare Madigan serotype distributions with the rest of the world (Table 1). Because most studies conducted in Mexico did not segregate serotype I into Ia and Ib, these studies were included in the systematic review (Table 3) but not in the final analysis (Table 1).

## Discussion

To determine the serotype of our Madigan population GBS isolates, we used a two-tiered PCR-based approach to identify type-specific capsular polysaccharides (CPS), epidemiologic markers used to classify serotypes according to prevalence for colonization and disease. The most common mechanism for identifying serotypes is serotype-specific latex agglutination analysis, but this method is prohibitively expensive when used on large patient populations [65]. Further, these tests have lower accuracy, and result in numerous NT isolates (Tables 2 and 3). In contrast, the polymerase chain reaction (PCR) based capsular typing techniques used in this study (i.e., RFLP analysis and multiplex PCR) are reproducible, specific, and easy to perform with fewer NT isolates reported [9,10]. We chose a two-tiered PCR approach, first using a restriction enzyme digest fingerprinting strategy followed by multiplex PCR for serotypes classified as NT by the RFLP methods [9,10]. There was 100% concordance between the two methods used to screen isolates, confirming accuracy of the classification scheme used in determining seroprevalences at Madigan.

The percentage of NT isolates reported in our study is significantly lower than that reported in international civilian GBS serotype surveillance studies using primarily the serotype-specific latex agglutination method (2-13%; Tables 1, 2, and 3) [65-67]. The 0.5% (n = 1 NT isolate) could result from an uncharacterized capsule [68], mutation in the capsular genes [69], or reversible nonencapsular phase variations [70]. Thus, the PCR-based method has proven an effective screening strategy for rapid and effective screening in a military population, and is anticipated to improve typing accuracy in larger cohorts as these methods become more widespread in surveillance studies.

To our knowledge, this is the first GBS serotype surveillance study targeting a population comprised solely of military beneficiaries. The primary mission of infectious disease research initiatives at military operated facilities is to identify and reduce the impact of infectious diseases affecting military populations. In accordance with this objective, this study reports a strikingly similar GBS distribution profile at Madigan relative to United States and Canadian studies (Tables 1 and 2). In comparing seroprevalences in the US with the Madigan cohort, our data suggest that seroprevalences have remained relatively stable in the United States over the past two decades since the emergence of serotype V in the mid-1990s (Table 2). Subtle regional differences in demographic makeup have been reported to affect GBS serodistribution within the same country [31]. Serotypic shifts have been reported in regional distributions in Korean study populations, for example, with a shift in prevalence of serotypes III and V depending on study site [42]. Regional data in early onset invasive GBS strains in neonates in the US indicate that GBS serotypes vary significantly according to study site, with more cases of serotype V reported in New Jersey and New York populations than in Florida, Texas, and Alabama [71]. However, combining study populations by geographical location led to a striking similarity between Madigan and the civilian sector in North America (Tables 1 and 2). It is currently unknown how rapidly seroconversion occurs in the general population. Our data plausibly reflect a stabilization of seroprevalence in the US over the past 15 years. However, very recent data indicate that serotype IV may be increasing in prevalence in the United States (8.4% of the 1160 patients enrolled from 2004 - 2008 in a multi-site United States study) [72].

Relative to global serodistribution studies, our statistical analysis indicates that North American populations have a higher representation of serotype Ia relative to other geographical locations (Tables 1 and 3). Since the emergence of serotype V in the general population, distributions of serotype proportion have shifted to accommodate the rise in serotype V prevalence in recent years in pregnant patients and neonates with invasive GBS disease [73]. Our global surveillance analysis suggests that Africa and South America have significantly greater serotype V representation relative to North American populations (Table 1).

The rate of GBS colonization rate at Madigan is comparable to the civilian-sector United States and Canada but differs from colonization rate reported in global surveillance studies (Tables 2 and 3). Data from the Emerging Pathogens Network places the overall GBS colonization incidence among U.S. women at approximately 10-30% [1,4]. At Madigan, GBS colonization rate was 18.3% during the study interval. East Asian countries report incidences as low as 0.3-5.9% [42], while African countries such as Zimbabwe report greater than 60% colonization in pregnant patients (Table 3) [74]. Ethnicity could account for some of these differences. African Americans and Hispanic Americans, for example, are GBS-colonized at higher rates than Caucasians [4,75]. Further, African American heritage is a recognized risk factor for early onset sepsis in neonates born to colonized mothers [1]. Finally, the mode of specimen collection (i.e., vaginal, rectal, or vaginal/rectal) has been reported to vary significantly depending on geographical location [1]. We recognize that mode of collection may bias estimates of global colonization and/or serodistribution.

Besides ethnicity and vertical transmission between colonized mother and neonate, colonization has also been associated with sexual contact and diet [22,76-79]. All modes are relevant to the Madigan population of highly mobile military beneficiaries and could account for the differences in GBS colonization rate observed between the Madigan cohort and the global studies.

Our systematic review and statistical comparison of the Madigan cohort with global epidemiological studies indicates variability in the geographical distribution of GBS serotypes (Tables 1 and 3). Studies with populations originating in Europe, Asia, Africa, Australia, and North and South America have reported significant differences in prevalence and emergence of novel serotypes over time (Table 3). When comparing Madigan seroprevalences with the global distributions, the Madigan cohort most closely resembles studies conducted in the continental United States and Canada (Table 2) [23].

Analysis of Tables 1 and 3 prompts the following conclusions regarding the seroprevalence of GBS in the Madigan cohort relative to international cohorts: (1) Ia is higher in North America relative to the rest of the world; (2) Ia and Ib are considerably higher in Mexico and South America; (3) III is lower in the Middle East and Eastern Europe than North America but higher in Africa and Australia/New Zealand; (4) IV is comparable across continents; (5) V is higher in Africa but much lower in Australia/New Zealand than North American populations; and (6) VI/VIII are most prevalent in Japan while (7) VII is least represented across the globe with occasional isolates reported in the Middle East/Eastern Europe and Asia. Limited population seroprevalance data are available for serotype IX given its very recent emergence in the general population [5,7]. Given that serotypes III and V (and to a lesser extent Ia) are most commonly associated with late onset neonatal illness, trends in locations such as Korea, Australia/New Zealand, and African countries with proportionally higher III seroprevalences underscore the need for continuing surveillance. An important caveat when interpreting the meta-data is the high NT rate reported in some studies (11.4-13.88%). The molecular methods more accurately assess serotype, potentially biasing the reporting of the serodistribution in the global comparative analyses.

Given the limited data available on GBS transmission [22,76-79], surveillance studies are warranted in military hospitals located in regions reporting unique seroprevalences. Serotypes typical of discrete geographical regions (i.e., VI and VIII) were less prevalent in our Madigan population (Table 1). Serotypes VIII and VI are most prevalent in Japan [41], a country which hosts numerous U.S. military bases and associated military hospitals. However, we report no incidences of VIII and only a small percentage of VI serotypes in our Madigan population. Similarly, neonatal GBS type IV disease has been reported as more prevalent in countries such as the United Arab Emirates than the United States [28]. Interestingly, our serodistribution data indicate that IV is the most stable serotype across all countries in the world, with no statistically significant differences reported (Table 1). Surveillance studies of seroconversion in female military operatives are warranted in military medical facilities located in Japan and the Middle East.

The implementation of CDC guidelines for anovaginal swab screening at 35-37 weeks gestation and subsequent intrapartum chemoprophylaxis in culture-positive individuals has reduced the incidence of GBS disease by 70% [1,8,80]. However, more than 25% of women now receive intrapartum antibiotics [81], raising public health concerns over the possibility of increasing antibiotic resistance of other common pathogens affecting neonatal health [2]. Further, although GBS readily succumbs to penicillin regimens, resistance is increasing in antibiotic alternatives such as clindamycin and erythromycin in women with penicillin allergy [82,83]. One strategy currently underway to combat this problem is the development of multivalent vaccines. Although vaccination efficacy and implementation feasibility remain controversial, understanding serotype distribution as a function of population is recognized as a critical component of vaccine development [6,84].

## Conclusions

In conclusion, the two-tiered molecular approach to GBS serotype analysis proved a viable strategy for assessing GBS serodistribution in the Madigan cohort, with fewer NT isolates than other methods employed in large population serodistribution studies. The ethnic diversity and geographical mobility of the United States military classify the military as a unique epidemiologic unit relative to US regional and global surveillance populations surveyed in our systematic review. However, the serodistributions reported in our study are remarkably comparable to those reported in civilian sector hospitals in the United States and Canada. Significant discrepancies exist between Madigan distributions and global epidemiology. Investigating seroprevalence in US military medical facilities abroad is necessary to determine whether military-specific populations will require specialized risk analysis for emerging GBS pathogens when alternatives to chemoprophylaxis come into clinical practice.

## Abbreviations

GBS: Group B *Streptococcus*; Madigan: Madigan Healthcare System; PCR: Polymerase Chain Reaction; RFLP: Restriction Fragment Length Polymorphism.

## Competing interests

The authors declare that they have no competing interests.

## Authors' contributions

DLI contributed to experimental design, study conception, pilot experiments, manuscript drafting, systematic review, and statistical analysis. WAJ, MJD, and JW assisted in data collection (PCR, glycerol stock preparation, and specimen banking), interpretation, and manuscript review. RRH conducted the meta-analysis, statistical interpretation, manuscript review. DT and MAW contributed to the systematic review, manuscript draft and revisions. STD contributed to experimental design, study conception, data collection and interpretation (PCR, glycerol stock preparation, specimen banking), manuscript drafting, systematic review, and statistical analysis. All authors read and approved the final manuscript.

## Funding

This project did not receive external financial support.

## Pre-publication history

The pre-publication history for this paper can be accessed here:

http://www.biomedcentral.com/1471-2334/10/336/prepub
